# Sanguinarine Improves Intestinal Health in Grass Carp Fed High-Fat Diets: Involvement of Antioxidant, Physical and Immune Barrier, and Intestinal Microbiota

**DOI:** 10.3390/antiox12071366

**Published:** 2023-06-29

**Authors:** Yong Shi, Yuanxiang Liu, Kai Xie, Junzhi Zhang, Ya Wang, Yi Hu, Lei Zhong

**Affiliations:** 1 Fisheries College, Hunan Agricultural University, Changsha 410128, China; shiyong@stu.hunau.edu.cn (Y.S.); 17373675292@stu.hunau.edu.cn (Y.L.); xk971006@stu.hunau.edu.cn (K.X.); zhjun123@hunau.edu.cn (J.Z.); 897772933@stu.hunau.edu.cn (Y.W.); 2 Hunan Engineering Research Center for Utilization of Characteristics of Aquatic Resources, Hunan Agricultural University, Changsha 410128, China

**Keywords:** Chinese herbal extract, *Ctenopharyngodon idellus*, high-fat diet, oxidative stress, intestinal barrier

## Abstract

An eight-week trial was conducted to investigate the effects of sanguinarine supplementation (600 μg and 1200 μg/kg) in high-fat (crude fat: 10%) diets (HF) on the intestinal physiological function of *Ctenopharyngodon idellus* (initial weight 50.21 ± 0.68 g), based on a basic diet (5% crude fat, CON), which were named HFLS and HFHS, respectively. The results showed that the HF diet significantly impaired the intestinal immune and physical barrier function, and disrupted the balance of the intestinal microbiota in grass carp. Compared to the HF diet, sanguinarine supplementation significantly improved the levels of serum C4, C3, AKP, IgA, and IgM, and enhanced the intestinal antioxidant capacity (*gr*, *CuZnsod*, *gpx4*, *cat*, *gsto*, and *nrf2* expression were significantly up-regulated). Sanguinarine significantly down-regulated the expression of *claudin-15* and up-regulated the expression of *claudin-b*, *claudin-c*, *occludin*, and *zo-1* by inhibiting MLCK signaling molecules. Additionally, sanguinarine significantly down-regulated the expression of *il-6*, *il-1β*, and *tnf-α* and up-regulated the expression of *il-10*, *tgf-β2*, and *tgf-β1* by inhibiting NF-κB signaling molecules, thereby alleviating intestinal inflammation caused by HF diets. Furthermore, compared to the HF diet, the abundance of Fusobacterium and *Cetobacterium* in the HFHS diet increased significantly, while the abundance of Firmicutes and *Streptococcus* showed the opposite trend. In conclusion, the HF diet had a negative impact on grass carp, while sanguinarine supplementation enhanced intestinal antioxidant ability, alleviated intestinal barrier damage, and ameliorated the homeostasis of the intestinal microbiota.

## 1. Introduction

Fat is one of the three major nutrients required by fish, providing essential cholesterol, phospholipids, and fatty acids necessary for maintaining health [1]. Numerous studies have demonstrated that increasing the dietary fat level appropriately can achieve a protein-saving effect [2]. This is primarily due to the body utilizing fat as an energy source, thereby reducing the decomposition of protein [3,4]. At the same time, due to the current global fish meal supply shortage and the rising price of plant and animal protein sources [5], high-energy diets are used on a large scale to provide the energy needed for the growth of aquatic animals and reduce farming costs [6,7]. Optimal dietary fat content has been shown to improve the growth and improve the feed conversion rate of *Micropterus salmoides* [8], *Lateolabrax japonicus* [9], and *Scophthalmus maximus* L. [10].

In aquatic animals, a moderate increase in dietary fat content can promote growth performance. However, the promotion effect does not exhibit a linear relationship with fat content. Studies have demonstrated that excessive fat in diets significantly reduces the growth of fish and has a significant negative impact on muscle quality [11,12,13]. High-fat diets induce intestinal inflammation by compromising the β-oxidation capacity of *Oreochromis niloticus* [14] and turbot [15], destroying the antioxidant system, and increasing the permeability of intestinal epithelial cells. Our previous studies on *Monopterus albus* also revealed that high-fat diets significantly reduced intestinal villus height and goblet cell numbers, reduce the expression of genes related to intestinal tight junction proteins, impair the physical barrier, and induce inflammation through the activation of NF-κB [16]. Moreover, dietary fat levels can affect the homeostasis of intestinal microbiota in aquatic animals [17]. In particular, long-term feeding of a high-fat diet can lead to an imbalance of intestinal microbiota diversity and structural disorders in zebrafish [18], rice field eel [19], and Nile tilapia [14], thereby affecting intestinal health. Additionally, the exogenous addition of functional additives to alleviate the intestinal physiological dysfunction caused by high-fat diets in aquatic animals represents a promising strategy.

Compared to other functional additives, Chinese herbal extracts have emerged as a research hotspot in aquaculture due to their advantages of small side effects, low toxicity, high efficiency, and cost-effectiveness. Sanguinarine (SAN), a quaternary benzo phenanthridine alkaloid, is mainly extracted from plants belonging to the *Papaveraceae* [20], including *Macleaya cordata (Willd.) R. Br*., *Argemone mexicana* L., *Chelidonium majus* L., and *Sanguinaria canadensis* L. [21]. Our laboratory has conducted numerous studies on SAN, which have demonstrated that dietary supplementation of SAN can relieve oxidative damage and inflammation induced by lipopolysaccharide and hydrogen peroxide in rice field eel [22,23]. Additionally, adding an appropriate amount of SAN to cottonseed and rapeseed meal diets has been shown to improve the intestinal health of grass carp [24]. Similar studies have also been conducted on Koi carp (*Cyprinus carpio*) [25]. Moreover, research in livestock and poultry has reported that dietary SAN can promote growth and regulates the cecal and intestinal microbiota of cattle [26], broiler chickens [27], and pigs [28]. These findings suggest that SAN, as an aquatic feed additive, exerts a protective effect on fish. However, it is worth noting that there is still a paucity of studies on the effects of SAN on intestinal health in fish, and the underlying regulatory mechanisms remain elusive.

*Ctenopharyngodon idellus*, a highly significant freshwater aquatic species of China, consistently ranks first in freshwater fish production every year. Therefore, grass carp was selected as the model species in this study to investigate whether SAN supplementation could relieve the detrimental effects of high-fat diets on fish. The evaluation encompassed comprehensive measurements of serum immune indexes and intestinal barrier function, including immune barrier, physical barrier, and microbial barrier. The purpose of this study was to provide theoretical support for the function and mechanism of sanguinarine as an aquatic feed additive and offer solutions to mitigate the negative impact caused by high-fat diets.

## 2. Materials and Methods

### 2.1. Animals Ethics Statement

All fish experiments were conducted according to the Guiding Principles for Care and Use of Laboratory Animals and were approved by the Hunan Agricultural University (No. 431639).

### 2.2. Feeding Trial and Experimental Diets

Four isonitrogenous diets were formulated as shown in Table 1: a control group (CON, 4.95% crude fat), a high-fat group (HF, 10.27% crude fat), an HF supplemented with 600 μg/kg SAN (HFLS), and an HF supplemented with 1200 μg/kg SAN (HFHS). The SAN (purity > 95%) used in this experiment was supplied by the Hunan Provincial Key Laboratory of Chinese Veterinary Medicine. All feed ingredients were crushed, passed through an 80-mesh sieve, and mixed gradually. Then soybean oil and water were added and thoroughly mixed. The diet pelleting mechanism was used to make 1.5 and 2.0 mm diets and then oven-dried at 55 °C for 10 h.

Grass carp (herbivorous fish, initial weight: 50.21 ± 0.68 g) were purchased from a local aquaculture plant (Changde, Hunan, China). After 24 h of fasting, 480 similar-sized and healthy fish were assigned to 12 cages randomly (2.0 × 2.0 × 2.0 m^3^, three cages per diet with 40 fish per cage). The fish were fed three times a day (7:00, 12:00, and 18:00), with each feeding amounting to 3–5% of their body weight, for a duration of 56 days. During the culture experiment, the water physicochemical parameters were maintained within the following ranges: dissolved oxygen levels between 6–8 mg/L, temperature ranging from 25–30 °C, and ammonia nitrogen < 0.4 mg/L.

### 2.3. Sampling

All fish were fasted for 24 h after breeding, after which eugenol (1:12,000) was used for anesthesia. The collection methods for serum were carried out following the procedures described in a previous study [29]. The midgut of three fish from each cage was removed, rinsed with sterile saline, and stored at −80℃ for detection of antioxidant enzyme activity, qRT-PCR, and western blot analysis. Additionally, the intestinal contents of six fish were collected from each cage, and the intestinal contents of three fish were combined into one sample and stored at −80 °C for microbiota analysis, so there were six samples in each group.

### 2.4. Proximate Composition

The proximate composition (ash, crude protein, and crude lipid) of diets was determined in accordance with previous studies [30].

### 2.5. Serum Immnue Parameters

Refer to the kit instructions (Nanjing Jiancheng Bioengineering Institute, Nanjing, China) to detect the ACP (acid phosphatase) and AKP (alkaline phosphatase) levels. Refer to the kit instructions (Zhejiang Yilikang Bioengineering Institute, Wenzhou, China) to measure the IgM (immunoglobulin M), IgA (immunoglobulin A), C4 (complement 4), and C3 (complement 3) contents.

### 2.6. Histopathological Analysis

Intestinal tissue was collected from three fish per replicate in the CON, HF, and HFHS groups. The tissue was fixed, paraffin-embedded, sectioned, and subjected to hematoxylin-eosin staining (Solarbio, Beijing, China) using the same procedures as described in previous studies [29]. Subsequently, the tissue sections were examined using an optical microscope (Olympus, Tokyo, Japan).

### 2.7. qRT-PCR Analysis

The intestinal tissue was placed in enzyme-free tubes containing RNA extract (TRIzol reagent, Carlsbad, CA, USA) and homogenized using a tissue grinder (Servicebio, Wuhan, China). After centrifugation, the supernatant was extracted by chloroform and precipitated by adding isopropanol. Finally, the precipitate was washed with anhydrous ethanol and dissolved in enzyme-free water. The purity and concentration of RNA were determined using a Nanodrop 2000 spectrophotometer. First-strand cDNA synthesis and the qRT-PCR procedure followed the methods described in a previous study [31]. All primers were composited by Shenggong Bioengineering Co., Ltd. (Shanghai, China) (Table 2). Amplification efficiencies of all genes were between 0.95 and 1.10. Values were quantified using the 2^−ΔΔCt^ method.

### 2.8. Western Blot

Intestinal tissue protein was extracted using RIPA lysate (Beyotime Biotechnology, Shanghai, China). The extraction method for intestinal nucleproteins followed the procedures described in a previous study [16]. The protein concentration was determined using the instructions provided with the kit instructions (Beyotime Biotechnology, Shanghai, China). The specific operation methods of gel electrophoresis, membrane transformation and antibody incubation refer to a previous study [16]. The primary antibodies were NF-κB p65, MLCK, and β-actin (rabbit, 1:1000, Affinity Biosciences, Cincinnati, OH, USA, Item No. AF5006, AF5314, and AF7018, respectively). The secondary antibodies were IgG (rabbit, 1:2000, S0001, Affinity Biosciences). Protein bands were detected using Beyoecl Plus (Beyotime Biotechnology, Shanghai, China) and visualized using the Genesys imaging system (Alcatel, Nanterre, France). The gray value was calculated using Image J software (v1.54, Bethesda, MD, USA).

### 2.9. Intestinal Microbiota

The CON, HF, and HFHS groups were analyzed for intestinal microbiota. Paired-end sequencing of DNA fragments was conducted using the Illumina platform. Sequence denoising and clustering were performed using DADA2 and Vsearch, respectively. Taxonomic annotation was carried out by QIIME software (http://www.mothur.org/wiki/Calculators; accessed on 10 January 2021). α-diversity was assessed by Chao1, Shannon, Simpson, Faith pd, Goods coverage, and Observed species index. Principal coordinate analysis was used for β-diversity based on weighted unifrac. The composition distribution at the phylum and genus level was visualized through statistical analysis of the feature table after removing singleton, and the analysis results were presented as stacked bar graphs.

### 2.10. Calculations and Statistical Analysis

The date was presented as mean ± SE. All statistical analyses were performed using SPSS 24.0. Significant differences were indicated by different superscripts (*p* < 0.05). The normality and homogeneity of the data were determined using the Shapiro-Wilk and Levene tests, respectively. One-way ANOVA and Duncan’s multiple comparisons of the means were used to examine the statistical significance in the event of equal variances and no significant deviation from normality.

## 3. Results

### 3.1. Immune Indices of Serum

No significant differences (*p* > 0.05) were shown in serum ACP activity among all groups (Table 3). The serum C4, C3, IgA, AKP, and IgM levels were significantly lower in an HF diet compared to a CON diet (*p* < 0.05). Compared with the HF diet, AKP, C3, C4, IgA, and IgM levels in HFHS diets were significantly higher (*p* < 0.05) and not significantly different from the CON diet.

### 3.2. Intestinal Antioxidant Enzyme Activities

**Figure 1:**
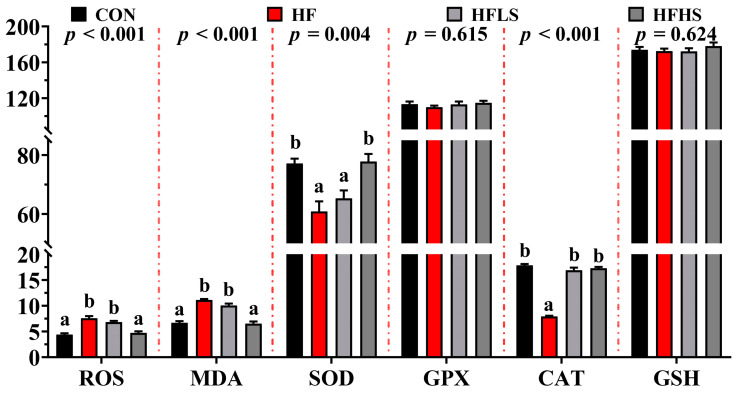
Effects of adding sanguinarine to a high-fat diet on intestinal antioxidant enzyme activities of grass carp. Reactive oxygen species (ROS, U/mg); Malondialdehyde (MDA, nmol/kg); Superoxide dismutase (SOD, U/mg); Glutathione peroxidase (GPX, U/mg); Catalase (CAT, U/mg); Glutathione (GSH, μmol/mg). Bars represent the mean ± SE. Significant differences are expressed by different superscripts (*p* < 0.05).

Figure 1 showed that no significant effects were found in GPX and GSH levels among all diets (*p* > 0.05). Compared with a CON diet, ROS and MDA contents of the intestine in an HF diet were significantly enhanced (*p* < 0.05), while SOD and CAT activities were significantly decreased. The CAT activity was significantly higher (*p* < 0.05) in an HFLS diet compared to an HF diet. ROS and MDA contents in an HFHS diet were significantly lower (*p* < 0.05) and CAT and SOD activities were significantly higher (*p* < 0.05) than that in the HF diet.

### 3.3. Intestinal Antioxidant-Related Gene Expression

**Figure 2:**
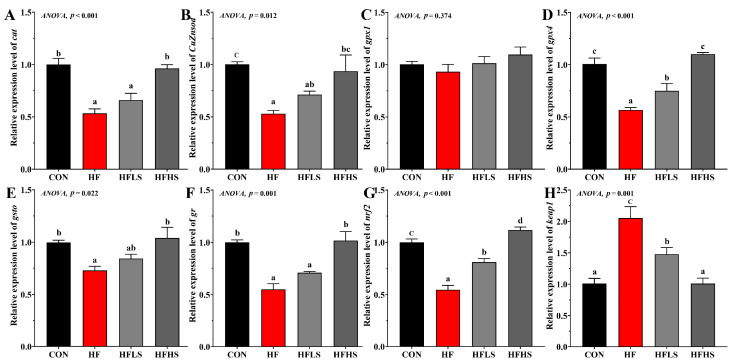
Effects of adding sanguinarine to a high-fat diet on intestinal antioxidant enzyme-related gene expression of grass carp. (**A**) *cat*; (**B**) *CuZnsod*; (**C**) *gpx1*; (**D**) *gpx4*; (**E**) *gsto*; (**F**) *gr*; (**G**) *nrf2*; (**H**) *keap1*. Bars represent the mean ± SE. Significant differences are expressed by different superscripts (*p* < 0.05).

Figure 2 showed that no significant effects were shown in *gpx1* expression among all diets (*p* > 0.05). Compared to a CON diet, *cat*, *CuZnsod*, *gpx4*, *gsto*, *gr*, and *nrf2* expression in the intestine were significantly lower in fish fed an HF diet, while *keap1* expression was opposite (*p* < 0.05). The mRNA expression of *gpx4* and *nrf2* were significantly higher (*p* < 0.05) in the HFLS group compared to the HF group. The HFHS group significantly enhanced *cat*, *CuZnsod*, *gpx4*, *gsto*, *gr*, and *nrf2* expression compared with the HF group, but reduced *keap1* expression.

### 3.4. Intestinal Morphology

**Figure 3:**
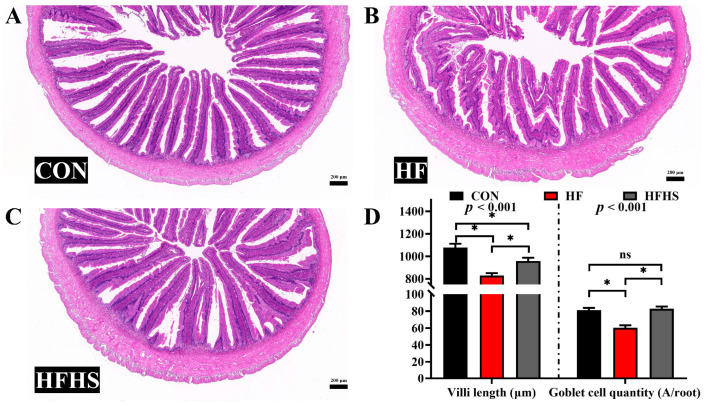
Effects of adding sanguinarine to a high-fat diet on intestinal morphology of grass carp. (**A**) control (CON) group; (**B**) high-fat (HF) group; (**C**) HF diet with 1200 μg/kg sanguinarine (HFHS) group; (**D**) intestinal villi length and goblet cell quantity. Bars represent the mean ± SE. *: *p* < 0.05, ns: *p* > 0.05.

Compared to the CON diet, villi length and goblet cell quantity of the intestine in the HF diet were significantly decreased (*p* < 0.05, Figure 3). The addition of 1200 μg/kg SAN to HF diets had the effect of increasing the villi length and goblet cell quantity compared to the HF diet (*p* < 0.05).

### 3.5. Intestinal Physical Barrier-Related Gene Expression and MLCK Protein Expression

**Figure 4:**
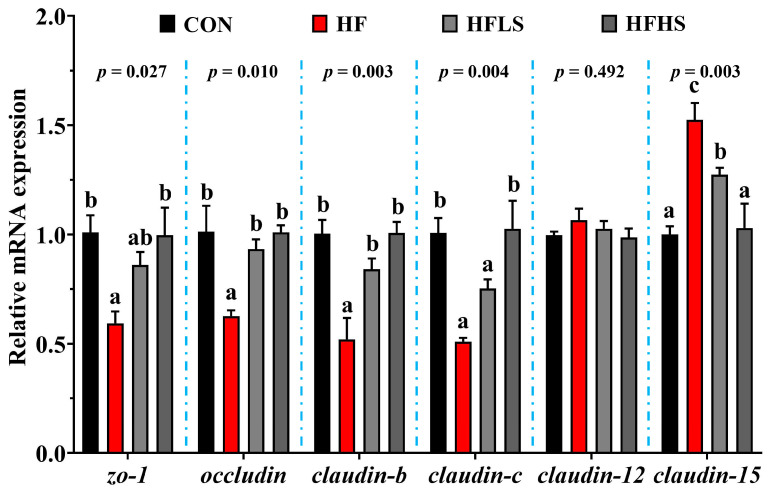
Effects of adding sanguinarine to a high-fat diet on intestinal physical barrier-related gene expression of grass carp. Bars represent the mean ± SE. Significant differences are expressed by different superscripts (*p* < 0.05).

Figure 4 showed that no statistical effect (*p* > 0.05) was found in *claudin-12* expression among all diets. Compared with the CON diet, the HF diet showed lower (*p* < 0.05) *claudin-c*, *zo-1*, *claudin-b*, and *occludin* expression, but a greater (*p* < 0.05) *claudin-15* expression. The mRNA expression of *occludin* and *claudin-b* was significantly higher (*p* < 0.05) in the HFLS group compared to the HF group. The mRNA expression of *claudin-15* was significantly lower (*p* < 0.05) in the HFLS group compared to the HF group. The HFHS group significantly enhanced *claudin-c*, *zo-1*, *claudin-b*, and *occludin* expression compared with the HF group, but reduced *claudin-15* expression (*p* < 0.05).

**Figure 5:**
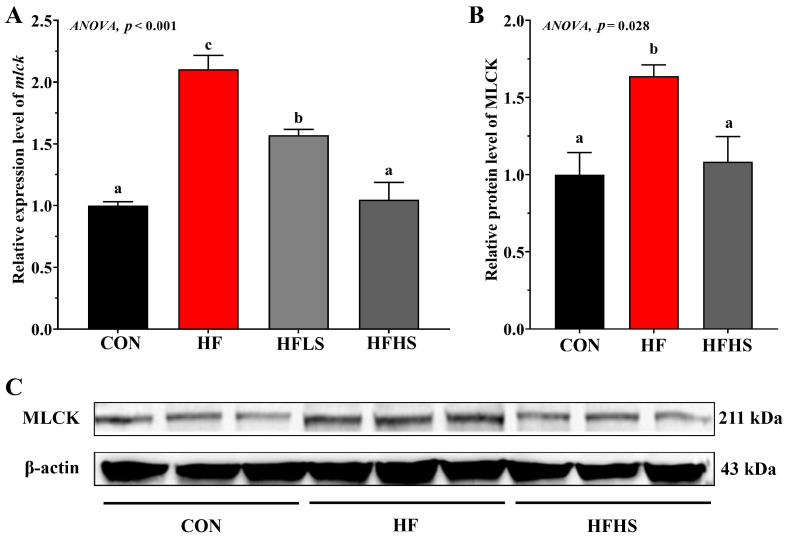
Effects of adding sanguinarine to a high-fat diet on MLCK gene expression and protein expression of grass carp. (**A**) relative gene expression level of *mlck*; (**B**) relative protein level of MLCK; (**C**) Blot of MLCK and β-actin. Bars represent the mean ± SE. Significant differences are expressed by different superscripts (*p* < 0.05).

Figure 5 showed that the HF diet showed significantly higher *mlck* expression and MLCK protein expression levels compared to the CON diet (*p* < 0.05). Adding 600 or 1200 μg/kg SAN to HF diets significantly down-regulated (*p* < 0.05) *mlck* expression compared to the HF diet. Compared with an HF diet, the MLCK protein expression level in an HFHS diet was significantly lower (*p* < 0.05) and was not significantly effective (*p* > 0.05) compared to that in a CON diet.

### 3.6. Intestinal Immune Barrier-Related Gene Expression and NF-κB Protein Expression

**Figure 6:**
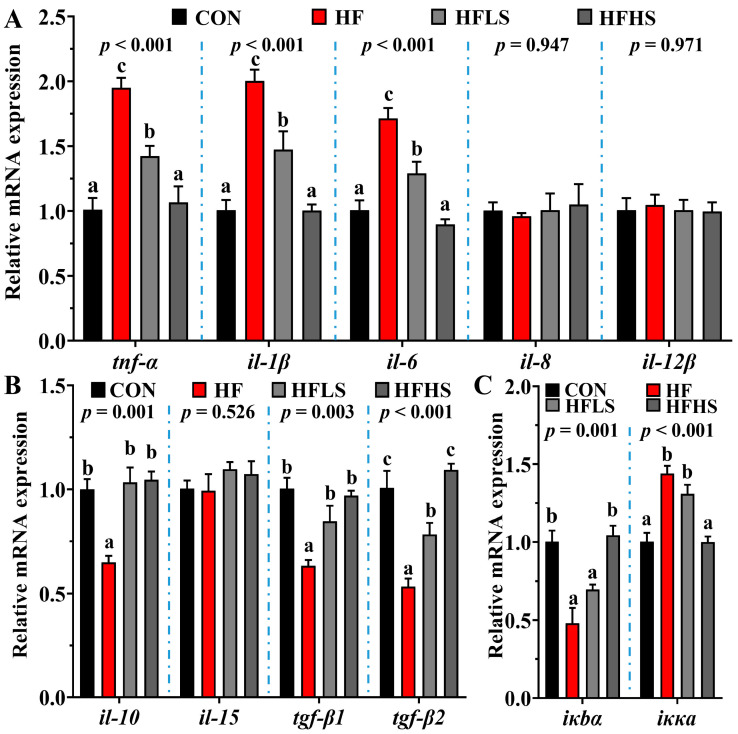
Influences of dietary mulberry leaf flavonoids on antioxidant relative genes expression in the livers of rice field eels fed a high-carbohydrate diet. (**A**) pro-inflammatory factor related expression; (**B**) anti-inflammatory factor related expression; (**C**) the expression of regulatory factors. Different letters show significant difference (*p* < 0.05).

Figure 6 showed that no statistical effect (*p* > 0.05) was found in *il-8*, *il-12β*, and *il-15* expression among all diets. Compared to a CON diet, *tgf-β2*, *tgf-β1*, *il-10*, and *iκbα* expression in the intestine were significantly lower in fish fed an HF diet, while *iκκα*, *il-6*, *tnf-α*, and *il-1β* expression were opposite (*p* < 0.05). Adding 600 or 1200 μg/kg SAN to HF diets significantly down-regulated (*p* < 0.05) *il-6*, *tnf-α*, and *il-1β* expression, up-regulated *tgf-β2*, *tgf-β1*, and *il-10* expression compared with the HF diet. Compared with an HF diet, HFLS diets significantly up-regulated *iκbα* expression, and down-regulated iκκα expression (*p* < 0.05).

**Figure 7:**
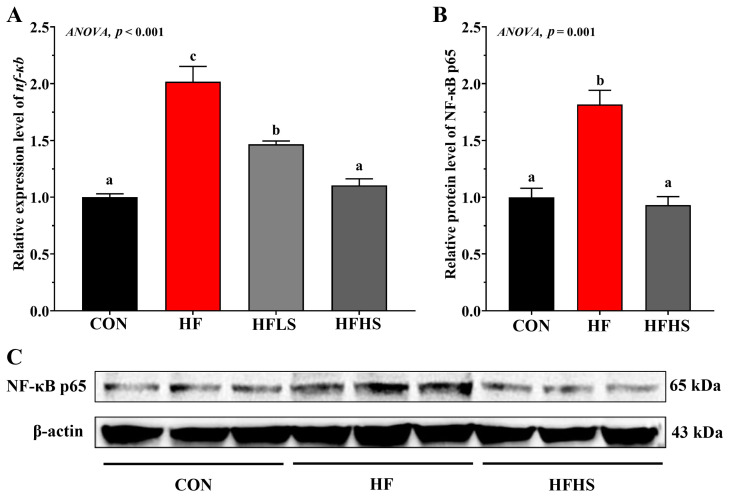
Effects of adding sanguinarine to a high-fat diet on NF-κB gene expression and protein expression of grass carp. (**A**) relative gene expression level of *nf-κb*; (**B**) relative protein level of NF-κB p65; (**C**) Blot of NF-κB p65 and β-actin. Bars represent the mean ± SE. Significant differences are expressed by different superscripts (*p* < 0.05).

Figure 7 showed that the HF diet showed significantly higher (*p* < 0.05) *nf-κb* expression and n-NF-κB p65 protein expression compared to the CON diet. Adding 600 or 1200 μg/kg SAN to HF diets significantly down-regulated *nf-κb* expression compared with the HF diet (*p* < 0.05). In addition, compared with an HF diet, the n-NF-κB p65 protein expression level in an HFHS diet was significantly lower (*p* < 0.05) and was not significantly effective (*p* > 0.05) compared to that in a CON diet.

### 3.7. Diversity Analysis of Intestinal Microbiota

**Figure 8:**
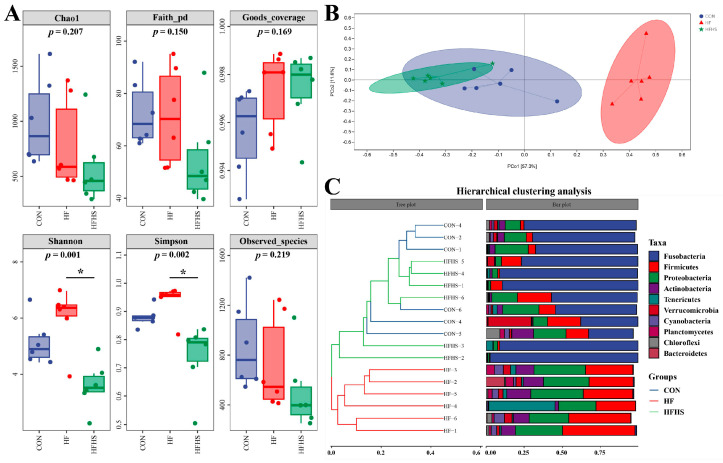
Effects of adding sanguinarine to a high-fat diet on α-, β-diversity, and evolutionary tree in intestinal microbiota of grass carp. (**A**) α-diversity; (**B**) β-diversity; (**C**) evolutionary tree. Bars represent the mean ± SE. * *p* < 0.05.

Figure 8A showed that no statistical effect was found in Chao1, Faith pd, Observed species, and Goods coverage indices among all diets (*p* > 0.05). Adding 1200 μg/kg SAN to HF diets significantly reduced the Simpson and Shannon compared to an HF diet (*p* < 0.05). Beta diversity and evolutionary tree analysis showed that the intestinal microbiota structure of CON and HFHS diets were similar, while the intestinal microbiota structure of the HF diet was far from that of CON and HFHS diets (Figure 8B,C).

### 3.8. Composition of Intestinal Microbiota

**Figure 9:**
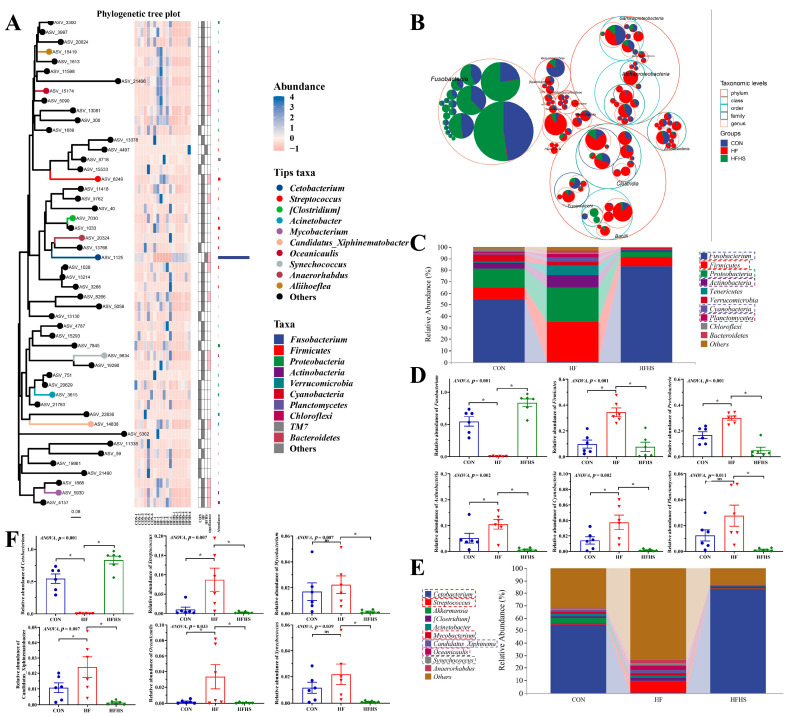
Effects of adding sanguinarine to high-fat diets on the microbial composition of grass carp. (**A**) phylogenetic tree plot; (**B**) classification hierarchy tree diagram (The largest circle represents phylum level, and the decreasing circle represents class, order, family, genus and species in descending order); (**C**) species abundance at the phylum level (Top 10); (**D**) showing significant variations of the relative abundance of intestinal microbiota at the phylum level; (**E**) species abundance at the genus level (Top 10); (**F**) showing significant variations of the relative abundance of intestinal microbiota at the genus level. Bars represent the mean ± SE. * *p* < 0.05.

The dominant bacteria in the intestine were Fusobacterium, Firmicutes, Proteobacteria, and Actinobacteria at the phylum level (Figure 9A–C). Compared with the CON and HFHS groups, the Firmicutes, Proteobacteria, Actinobacteria, and Cyanobacteria abundances in the HF group were significantly enhanced in the top ten at the phylum level, while the Fusobacterium abundance was opposite (*p* < 0.05) (Figure 9C,D). In the top ten at the genus level, it was found that the abundances of *Streptococcus*, *Mycobacterium*, *Candidatus_Xiphinematobacter*, and *Oceanicaulis* were significantly enhanced in an HF diet compared to CON and HFHS diets, while the *Cetobacterium* abundance (*p* < 0.05, Figure 9E,F) was not. 

### 3.9. Multidimensional Correlation Analysis

**Figure 10:**
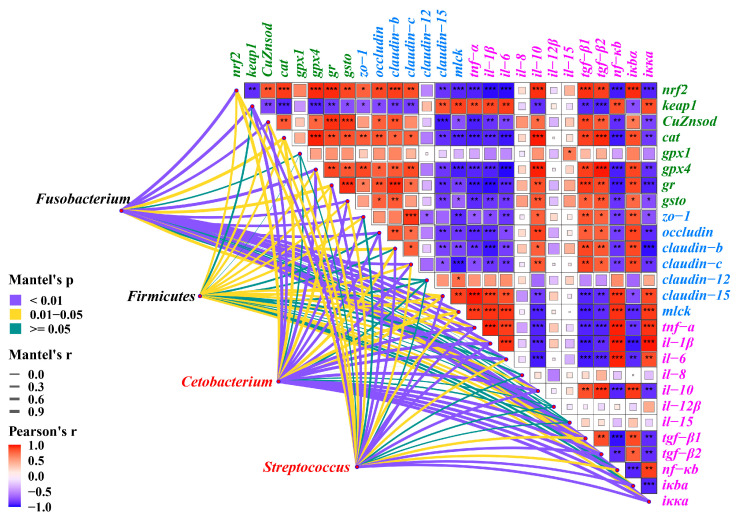
Effects of adding sanguinarine to high-fat diets on the microbial composition of grass carp. Bars represent the mean ± SE. * *p* < 0.05, ** *p* < 0.01, *** *p* < 0.001.

*nrf2* expression has a significant positive correlation with the gene expression levels of *CuZnsod*, *cat*, *gpx4*, *gr*, and *gsto*, while a significant negative correlation with the gene expression level of keap1 (*p* < 0.05, Figure 10). *mlck* expression was significantly negatively correlated with *zo-1*, *claudin-c*, *claudin-b*, and *occludin* expression, and significantly positively correlated with *claudin-12* and *claudin-15* expression (*p* < 0.05). *nf-κb* expression was positively correlated with *iκκα*, *il-6*, *tnf-α*, and *il-1β* expression, and negatively correlated with *il-10*, *tgf-β2*, *tgf-β1*, and *iκbα* expression (*p* < 0.05). *Furthermore*, Fusobacterium, Firmicutes, *Cetobacterium*, and *Streptococcus* were significantly correlated with the expression of genes related to intestinal antioxidants, physical barriers, and immune barriers (*p* < 0.05).

## 4. Discussion

The humoral immunity of aquatic animals is an essential component of non-specific immunity [32]. AKP and ACP in the serum are important protective lysosomal enzymes in fish, which can indirectly reflect the physiological status [33]. IgM is the primary antibody secreted by aquatic animals in response to antigen stimulation [34], and the complement system is also a crucial defense against pathogens [35]. This study showed that long-term intake of an HF diet significantly reduced the immunity of grass carp, specifically leading to a noticeable decrease in serum levels of C3, C4, IgA, IgM, and AKP. Similar results were observed in rice field eels [16], *Megalobrama amblycephala* [36], and *Acanthopagrus schlegelii* [37]. Currently, there are more and more reports about adding plant extracts to diets to improve the immune function of aquatic animals [38]. To further investigate whether SAN extracted from *Macleaya cordata* (Willd.) R. Br. can alleviate the immune decline in grass carp caused by a high-fat diet, a study was conducted. The study showed that adding 1200 μg/kg SAN prevents the diminution of serum IgM, IgA, C3, C4, and AKP levels induced by the HFD diet. Our previous studies also found that dietary SAN can alleviate the immune damage induced by LPS by improving the non-specific immune ability of rice field eels [22]. A similar effect has been observed in livestock [39]. These findings suggest that sanguinarine or its metabolites may activate non-specific immunity, thereby stimulating an increase in C3, C4, IgM, IgA, and AKP levels to protect fish from damage. However, the underlying mechanism requires further investigation.

The oxidative homeostasis in aquatic animals is easily disrupted under long-term stress, resulting in oxidative stress and further impairing tissue function [40]. Within the intestine, its antioxidant capacity is crucial for maintaining the structural integrity of intestinal epithelial cells [41]. MDA and ROS serve as biomarkers of oxidative damage [31]. This study showed that long-term feeding of high-fat diets significantly increased the levels of ROS and MDA in the intestine of grass carp, indicating that high-fat diets can induce oxidative stress, which is consistent with our previous study with rice field eels [16]. In aquatic animals, non-enzymatic antioxidant substances (GSH) and antioxidant enzymes (CAT, GPX, and SOD) play an important role in the process of reducing oxidative damage [42]. The activity of the above antioxidant enzymes is controlled by the corresponding genes. For example, the expression levels of *cat*, *CuZnsod*, *gsto*, *gr*, and *gpx4* genes are increased, and the corresponding activities of CAT, SOD, GST, GR, and GPX are increased, thus enhancing the antioxidant capacity [23]. This study showed that the intestinal CAT and SOD activities and antioxidant-related genes (*cat*, *CuZnsod*, *gsto*, *gr*, and *gpx4*) expression were significantly reduced after feeding high-fat diets. Similar findings were observed in juvenile *Oreochromis niloticus*, where a high-fat diet led to reduced activities of antioxidant enzymes and down-regulated expression of their related genes [43]. The main reason is the accumulation of fat, which leads to an increase in fat peroxidation, thus inducing oxidative stress [44]. Other studies have demonstrated that a high-fat diet can induce intestinal oxidative stress by inhibiting *nrf2* expression and down-regulating the expression of antioxidant enzyme genes [18], which aligns with the results of this experiment.

Based on our previous studies on SAN, it was found that the addition of an appropriate amount of SAN to the diet can up-regulate the activities of antioxidant enzymes through the Nrf2-Keap1, effectively inhibiting oxidative stress of rice field eel induced by hydrogen peroxide [23]. SAN has also demonstrated strong antioxidant capacity in livestock [45,46]. Therefore, our investigation aimed to assess whether adding sanguinarine to high-fat diets could alleviate oxidative damage. The results revealed that the supplementation of 1200 μg/kg SAN in a high-fat diet exerted a significant antioxidant effect. This effect was primarily achieved by up-regulating the expression of *cat*, *CuZnsod*, *gpx4*, *gsto*, *gr*, and *nrf2*, while down-regulating the expression of *keap1*. Consequently, the activities of antioxidant enzymes were enhanced, and the ROS and MDA contents were decreased. Previous studies have also demonstrated that SAN can enhance antioxidant function by activating Nrf2 [47]. Therefore, this study provides further confirmation of the potential of SAN as a plant additive in diets to promote the antioxidant function of fish.

In aquatic animals, the intestinal physical barrier is not only damaged by oxidative stress [48] but also depends on tight junctions between intestinal epithelial cells [49]. Tight junction proteins can be classified into two main categories: transmembrane proteins (such as Occludin and Claudins) and cytoplasmic proteins (such as ZOs), which play an important role in maintaining the integrity of fish intercellular structures and preventing foreign microorganisms and pathogens from entering the cells [50]. Studies in fish have found that upregulation of *occludin*, *ZO-1*, *claudin-b*, and *claudin-c* mRNA levels stabilizes intercellular structural integrity, while upregulation of *claudin-12* and *claudin-15* mRNA levels disrupts the structural integrity of cells [51]. Goblet cells, located on intestinal villi, play a crucial role in maintaining the integrity of the intestinal mucosal by secreting mucin glycoproteins [52]. Some studies have demonstrated that high-fat diets can increase intestinal permeability in mice by regulating the expression of intestinal tight junction proteins, thereby compromising the function of the intestinal physical barrier [53,54]. In this study, long-term feeding of high-fat diets significantly down-regulated *zo-1*, *occludin*, *claudin-b*, and *claudin-c* expression, and *claudin-15* expression was opposite and reduced the number of goblet cells. Furthermore, studies also have reported that high-fat diets can significantly reduce the height of intestinal villi and cause irregularities in their structure [55]. These findings indicate that high-fat diets can impair the physical barrier function of the intestine through changes in the expression of tight junction protein-related genes and alterations in the structure of intestinal villi.

Several studies have indicated that the addition of *Macleaya cordata* extract (the active ingredient that contains SAN) to the diet can improve intestinal *occludin* and *zo-1* expression in laying hens, and enhance the intestinal physical barrier function [56]. In the present study, adding SAN to high-fat diets can up-regulate *occludin*, *zo-1*, *claudin-c*, and *claudin-b* expression, and increase the height of intestinal villus and the number of goblet cells, to alleviate intestinal physical barrier dysfunction induced by high-fat diets. This result is similar to our previous results of adding sanguinarine to high cottonseed and rapeseed meal diets for grass carp [24]. SAN was also shown to promote the proliferation of intestinal epithelial cells in porcine [57]. Some studies have shown that the tight junctions between intestinal cells are mainly affected by MLCK signaling molecules [58]. MLCK activation promotes myosin II to regulate light chain phosphorylation, stimulating actin/myosin binding and its subsequent contraction, which ultimately leads to the opening of tight junctions and the formation of a more permeable paracellular pathway since actin is linked to tight junctions [58]. Studies have reported that MLCK is negatively correlated with the expression levels of *claudin-c*, *zo-1*, and *claudin-b*, and positively correlated with the expression levels of *claudin-15* and *claudin-12* [59]. By activating MLCK signaling molecules, *claudin-15* and *claudin-12* expression is downregulated, while *claudin-c*, *zo-1*, and *claudin-b* expression are upregulated Early reports of SAN as a potent MLCK inhibitor have been reported [60], but has not been reported in aquatic animals. In this study, the addition of SAN to the high-fat diet significantly inhibited the expression of intestinal MLCK protein, thereby regulating tight-junction protein-related genes, and restoring the physical barrier function. In addition, this study found through correlation studies, MLCK is positively correlated with the expression levels of *claudin-15* and *claudin-12*, and negatively correlated with the expression levels of *occludin*, *claudin-c*, *zo-1*, and *claudin-b, but the specific regulatory mechanism needs further study and discovery*. Based on the results of these studies, SAN can alleviate the impairment of intestinal physical barrier function in grass carp caused by high-fat diets through MLCK signaling molecules, and further enrich the mechanism of SAN in alleviating barrier function.

In addition to the physical barrier, the immune barrier is also crucial for maintaining the intestinal health of fish [59]. In aquatic animals, a reduction in anti-inflammatory cytokines (such as *tgf-β* and *il-10*) and an increase in pro-inflammatory cytokines (such as *tnf-α*, and *il-6*, *il-1β*) aggravate inflammatory responses [31]. Numerous studies have demonstrated that feeding on high-fat diets can up-regulate the expression of pro-inflammatory factors (*tnf-α* and *il-1β*, etc.) in various fish species, including tilapia [61], black seabream [37], and blunt snout bream [62]. In this study, grass carp fed with high-fat diets showed intestinal inflammation, which was specifically manifested in the increase of *tnf-α, il-6*, and *il-1β* expression and reduction of *tgf-β1*, *il-10*, and *tgf-β2* expression. Studies on SAN have shown that the addition of SAN to diets effectively inhibits the enrichment of *il-6* and *il-1β* in Koi carp, and enhances resistance against pathogenic bacteria invasion [25]. Previous studies have also demonstrated that dietary supplementation of SAN can relieve intestinal inflammation in rice field eel induced by hydrogen peroxide [22]. This study also found that the addition of SAN to a high-fat diet effectively alleviated the inflammatory response, primarily characterized by the up-regulation of anti-inflammatory factors such as *tgf-β1*, *tgf-β2*, and *il-10*, and the down-regulation of pro-inflammatory factors such as *tnf-α*, *il-6*, and *il-1β*.

NF-κB is an important transcription factor that regulates cytokines [63]. The IKK complex promotes the degradation of the NF-κB inhibitor IκBα, leading to the nuclear displacement of NF-κB and enhanced the expression of pro-inflammatory cytokines [64]. Previous studies have demonstrated that long-term high-fat diets can activate NF-κB and trigger inflammatory responses in aquatic animals, such as golden pompano [65], Nile tilapia [66], and zebrafish [67]. SAN has been proven to be an effective inhibitor of NF-κB [20]. The results of in vitro experiments also proved that SAN can alleviate LPS-induced inflammation in H9c2 cardiomyocytes by inhibiting NF-κB/TLR signaling [68]. This study also showed that SAN significantly inhibited the protein expression of NF-κB, significantly down-regulated *iκκα* expression, and up-regulated *iκbα* expression. Studies have shown that up-regulation of *iκbα* gene expression and down-regulation of *iκκα* gene expression can promote anti-inflammatory function [51]. In this study, it was also found through correlation analysis that the expression trend of *iκbα* was significantly negatively correlated with *tnf-α*, *il-6*, *il-1β*, *nf-κb*, and *iκκα*, and was significantly positively correlated with the expression trend of *tgf-β1*, *tgf-β2*, and *il-10*. These results indicated that SAN can alleviate the inflammatory response of grass carp induced by high-fat diets by inhibiting the NF-κB signaling pathway.

It is well known that intestinal health is closely related to the composition of intestinal microbiota and its metabolites, which impact host metabolism, immunity, physiology, and nutrition [69]. Recent studies have shown that a high-fat diet, induced inflammation, and impaired intestinal barrier function in animals have an important relationship with changes in the structure of the intestinal microbiota [70]. In this study, the high-fat diet exhibited a trend of increasing Shannon and Simpson index, and the intestinal microbiota structure of grass carp in the high-fat group showed significant changes according to PCoA analysis. Similar findings were observed in zebrafish, where the high-fat group showed an increased diversity index and an observable change in the structure of intestinal microbiota [18]. The increased diversity of the intestinal microbiota may be related to the presence of pathogenic bacteria species, although further study and analysis are required. Following the addition of 1200 μg/kg SAN, the microbiota structure and diversity index of grass carp resembled those of the CON group, indicating that SAN positively contributed to the homeostasis of the intestinal microbiota.

Fusobacterium, Firmicutes, Proteobacteria, and Actinobacteria were identified as the predominant bacteria in the intestinal microbiota of grass carp, which is consistent with previous research [71]. Studies have confirmed that a high-fat diet can improve Firmicutes abundance [72]. This study also found that a high-fat diet observably decreased the Fusobacterium abundance and increased the Firmicutes abundance. Furthermore, at the genus level, this study also revealed that a high-fat diet observably reduced the *Cetobacterium* abundance and increased the *Streptococcus* abundance. Previous studies have shown that the Fusobacterium and *Cetobacterium* abundances increases are beneficial for the health of fish [73,74].

*Cetobacterium* is also thought to produce acetate and butyrate, which can help improve intestinal health [75]. Therefore, these results further confirmed that a high-fat diet can cause intestinal microbiota disturbance. A growing number of studies have demonstrated that plant extracts can alleviate oxidative stress and inflammatory responses by influencing the composition of the intestinal microbiota [76]. In livestock, SAN has been shown to improve the growth of piglets [45] and broilers [27] by promoting intestinal microbiota homeostasis. Similar results were observed in Koi carp (*Cryprinus carpiod*), where the addition of SAN to diets reduced harmful bacteria and increased beneficial bacteria, leading to enhanced immunity [25]. These results showed that adding SAN to high-fat diets significantly improved the abundance of Fusobacterium and reduced the abundance of Firmicutes. At the genus level, SAN significantly increased the abundance of *Cetobacterium* and effectively reduced the abundance of *Streptococcus*.

In addition, correlation analysis showed that the changes in the abundances of Fusobacterium, Firmicutes, *Cetobacterium*, and *Streptococcus* were correlated with the expression of related genes in the intestine (including antioxidant, physical barrier, and immune barrier). Specifically, Fusobacterium and *Cetobacterium* showed a significant positive correlation with the expression of antioxidant enzymes and anti-inflammatory factor-related genes in the intestine. On the other hand, they exhibited a significant negative correlation with the expression of pro-inflammatory factor-related genes. In contrast, Firmicutes and Streptococcus displayed an opposite trend in terms of their correlation with the aforementioned gene expressions. These results highlight the crucial role of Fusobacterium, Firmicutes, *Cetobacterium*, and *Streptococcus* in intestinal health. Therefore, it can be further confirmed that the alleviation of high-fat diets induced intestinal injury by SAN is closely related to its regulation of intestinal microbiota.

## 5. Conclusions

The study suggested that the high-fat diet significantly reduced the immunity of grass carp and resulted in intestinal dysfunction, mainly including antioxidant, physical barrier, immune barrier, and microbiota barrier. The addition of 1200 μg/kg sanguinarine in the high-fat diet enhanced the immunity of grass carp and alleviated the damage of the intestinal physical and immune barriers by regulating the MLCK and NF-κB signaling molecules. Furthermore, sanguinarine improved intestinal microbiota homeostasis by regulating the structure of the intestinal microbiota.

## Figures and Tables

**Table 1:** Formulation and calculated chemical compositions of the experimental diet.

	CON	HF	HFLS	HFHS
Fish meal (%)	6	6	6	6
Soybean meal (%)	26	26	26	26
Cottonseed meal (%)	10	10	10	10
Rapeseed meal (%)	15	15	15	15
Flour (%)	27	27	27	27
DDGS (%)	4	4	4	4
Microcrystalline cellulose (%)	6.06	1.06	1.06	1.06
Choline chloride (%)	0.2	0.2	0.2	0.2
Soybean oil (%)	3.2	8.2	8.2	8.2
Ca(H_2_PO_4_)_2_ (%)	1.5	1.5	1.5	1.5
Premix ^a^ (%)	1	1	1	1
Mould inhibitor (%)	0.03	0.03	0.03	0.03
Antioxidants (%)	0.01	0.01	0.01	0.01
Sanguinarine (μg/kg)	0	0	600	1200
Proximate composition (%)				
Saturated fatty acids	22.48	17.35	17.19	17.24
Unsaturated fatty acids	73.56	78.17	78.26	78.34
Crude protein	31.72	31.32	31.98	31.75
Crude lipid	4.95	10.27	10.36	10.09
Ash	6.42	6.35	6.42	6.64
Carbohydrate	34.27	34.32	34.18	34.26
Crude fiber	12.48	7.17	7.26	7.21

^a^ Provided by MGO Ter Bio-Tech (Qingdao, Shandong, China).

**Table 2:** List of primers used in this study.

Gene	Forward Sequences (5′→3′)	Reverse Sequences (5′→3′)	Accession Number
*nrf2*	CTGGACGAGGAGACTGGA	ATCTGTGGTAGGTGGAAC	KF733814
*keap1*	TTCCACGCCCTCCTCAA	TGTACCCTCCCGCTATG	KF811013
*gpx1*	GGGCTGGTTATTCTGGGC	AGGCGATGTCATTCCTGTTC	EU828796
*gpx4*	TACGCTGAGAGAGGTTTACACAT	CTTTTCCATTGGGTTGTTCC	KU255598
*gr*	GTGTCCAACTTCTCCTGTG	ACTCTGGGGTCCAAAACG	JX854448
*cat*	GAAGTTCTACACCGATGAGG	CCAGAAATCCCAAACCAT	FJ560431
*CuZnsod*	CGCACTTCAACCCTTACA	ACTTTCCTCATTGCCTCC	GU901214
*gsto*	GGTGCTCAATGCCAAGGGAA	CTCAAACGGGTCGGATGGAA	KT757314
*zo-1*	CGGTGTCTTCGTAGTCGG	CAGTTGGTTTGGGTTTCAG	KJ000055
*occludin*	TATCTGTATCACTACTGCGTCG	CATTCACCCAATCCTCCA	KF193855
*claudin-b*	GAGGGAATCTGGATGAGC	ATGGCAATGATGGTGAGA	KF193860
*claudin-c*	GAGGGAATCTGGATGAGC	CTGTTATGAAAGCGGCAC	KF193859
*claudin-12*	CCCTGAAGTGCCCACAA	GCGTATGTCACGGGAGAA	KF998571
*claudin-15*	TGCTTTATTTCTTGGCTTTC	CTCGTACAGGGTTGAGGTG	KF193857
*mlck*	GAAGGTCAGGGCATCTCA	GGGTCGGGCTTATCTACT	KM279719
*tnf-α*	CGCTGCTGTCTGCTTCAC	CCTGGTCCTGGTTCACTC	HQ696609
*il-1β*	AGAGTTTGGTGAAGAAGAGG	TTATTGTGGTTACGCTGGA	JQ692172
*il-6*	CAGCAGAATGGGGGAGTTATC	CTCGCAGAGTCTTGACATCCTT	KC535507.1
*il-8*	ATGAGTCTTAGAGGTCTGGGT	ACAGTGAGGGCTAGGAGGG	JN663841
*il-10*	AATCCCTTTGATTTTGCC	GTGCCTTATCCTACAGTATGTG	HQ388294
*il-12β*	ACAAAGATGAAAAACTGGAGGC	GTGTGTGGTTTAGGTAGGAGCC	KF944668.1
*il-15*	CCTTCCAACAATCTCGCTTC	AACACATCTTCCAGTTCTCCTT	KT445872
*tgf-β1*	TTGGGACTTGTGCTCTAT	AGTTCTGCTGGGATGTTT	EU099588
*nf-κb*	GAAGAAGGATGTGGGAGATG	TGTTGTCGTAGATGGGCTGAG	KJ526214
*iκbα*	TCTTGCCATTATTCACGAGG	TGTTACCACAGTCATCCACCA	KJ125069
*iκκα*	GGCTACGCCAAAGACCTG	CGGACCTCGCCATTCATA	KM279718
*iκκβ*	GTGGCGGTGGATTATTGG	GCACGGGTTGCCAGTTTG	KP125491
*β-actin*	GGCTGTGCTGTCCCTGTA	GGGCATAACCCTCGTAGAT	M25013

**Table 3:** Effects of adding sanguinarine to a high-fat diet on serum immune indices of grass carp.

Index	CON	HF	HFLS	HFHS	*p*-Value
ACP ^1^ (King’s unit/1000 mL)	18.29 ± 0.12	18.08 ± 0.19	18.16 ± 0.16	18.24 ± 0.24	0.853
AKP ^2^ (King’s unit/1000 mL)	14.88 ± 0.54 ^b^	11.58 ± 0.47 ^a^	12.79 ± 0.37 ^a^	14.40 ± 0.56 ^b^	0.005
C3 ^3^ (g/L)	0.39 ± 0.04 ^bc^	0.24 ± 0.02 ^a^	0.34 ± 0.02 ^b^	0.45 ± 0.03 ^c^	0.005
C4 ^4^ (g/L)	0.14 ± 0.01 ^c^	0.06 ± 0.00 ^a^	0.09 ± 0.01 ^b^	0.14 ± 0.00 ^c^	<0.001
IgA ^5^ (g/L)	0.35 ± 0.02 ^c^	0.14 ± 0.01 ^a^	0.21 ± 0.02 ^b^	0.31 ± 0.03 ^c^	<0.001
IgM ^6^ (g/L)	1.32 ± 0.08 ^b^	0.70 ± 0.04 ^a^	0.81 ± 0.03 ^a^	1.24 ± 0.10 ^b^	0.001

Note: Values with different letter superscripts are significantly different (*p* < 0.05). ^1^ ACP, Acid phosphatase. ^2^ AKP, Alkaline phosphatase. ^3^ C3, Complement 3. ^4^ C4, Complement 4. ^5^ IgA, Immunoglobulin A. ^6^ IgM, Immunoglobulin M.

## Data Availability

All data generated or analyzed during this study are included in this published article.
